# Personal wellbeing in posttraumatic stress disorder (PTSD): association with PTSD symptoms during and following treatment

**DOI:** 10.1186/s40359-018-0219-2

**Published:** 2018-03-02

**Authors:** David Berle, Dominic Hilbrink, Clare Russell-Williams, Rachael Kiely, Laura Hardaker, Natasha Garwood, Anne Gilchrist, Zachary Steel

**Affiliations:** 1 Discipline of Clinical Psychology, Graduate School of Health, University of Technology Sydney, Building 7, 67 Thomas Street, Ultimo, NSW 2007 Australia; 2 School of Psychiatry, UNSW, Sydney, Australia; 3 St John of God Health Care, Richmond Hospital, Richmond, Australia

**Keywords:** Wellbeing, Trauma, Quality of life, Inpatient, Treatment, Posttraumatic stress disorder, PTSD

## Abstract

**Background:**

It remains unclear to what extent treatment-related gains in posttraumatic stress disorder (PTSD) symptoms translate to improvements in broader domains of personal wellbeing, such as community connectedness, life achievement and security. We sought to determine whether: 1. personal wellbeing improves during the course of a treatment program and 2. changes in core symptom domains (PTSD, anxiety and depression) were associated with improvements in overall personal wellbeing.

**Methods:**

Participants (*N* = 124) completed the PTSD Checklist, the Depression and Anxiety Stress Scales and the Personal Wellbeing Index at the start and end of a 4-week Trauma Focused CBT residential program, as well as 3- and 9-months post-treatment.

**Results:**

Personal wellbeing improved significantly across the 9-months of the study. Generalised estimating equations analyses indicated that (older) age and improvements in PTSD and depressive symptoms were independent predictors of personal wellbeing across time.

**Conclusions:**

Although personal wellbeing improved in tandem with PTSD symptoms, the magnitude of improvement was small. These findings highlight a need to better understand how improvements in personal wellbeing can be optimised following PTSD treatment.

**Electronic supplementary material:**

The online version of this article (10.1186/s40359-018-0219-2) contains supplementary material, which is available to authorized users.

## Background

Individuals with posttraumatic stress disorder (PTSD) report high levels of dissatisfaction across multiple life domains including physical health [1] and social and occupational functioning [2]. It is generally assumed that evidence-based interventions for PTSD, if effective, should also lead to broader improvements in life satisfaction [3].

Evidence suggests that self-rated quality of life in those with PTSD improves in tandem with symptom improvement during psychological [3] and pharmacological [4] treatment. However, many of these studies focused on health-related quality of life (e.g., physical and mental health quality of life as assessed by measures such as the Short Form Health Survey [5]). Notwithstanding the overlap with symptom measures, a reliance on disability-focused measures may have perpetuated an assumption that quality of life and wellbeing are synonymous with the absence of impairment. There thus remains a need for a more comprehensive assessment of quality of life in relation to PTSD treatment if the full benefits of PTSD treatment are to be adequately understood.

Personal wellbeing refers to the subjective dimension of quality of life [6]. In addition to the physical and mental health domains of quality of life, the notion of personal wellbeing captures a broader range of dimensions including perceptions of one’s standard of living, life achievement, quality of personal relationships, perceived safety, community engagement and future security [6]. Furthermore, the concept of personal wellbeing incorporates the possibility that an individual might thrive rather than simply lack disability.

Overall life satisfaction is a similar concept to that of personal wellbeing and appears to be relatively low in individuals with PTSD [7]. However, there remains a relative absence of research that has used measures capturing a broad range of domains of personal wellbeing in treatment people receiving treatment for PTSD (such as relationship satisfaction, perceived safety, etc). For instance, the few studies that have investigated changes in either life satisfaction or personal wellbeing during PTSD treatment, have typically focused only on one specific domain, such as spiritual wellbeing (e.g., [8]). An exception was a treatment trial of venlafaxine versus sertraline in addition to psychotherapy for refugees with PTSD, which reported small to medium-size improvements in wellbeing from pre to posttreatment, but did not include a follow-up assessment to determine whether the benefits persisted [9]. Investigation of whether improvements in perceived wellbeing persist is important for ensuring that changes in perceived wellbeing are reliable and remain once individuals complete treatment programs and return to their home environments.

Improving our understanding of these processes has the potential to provide a deeper understanding of the broader benefits of PTSD treatment beyond symptoms alone. For example, even though there is increasing evidence to suggest that symptomatic improvement persists following treatment, it remains unclear whether this also applies for any improvements in personal wellbeing. There are various pathways by which psychological therapies could affect changes in wellbeing, one being an increased sense of self-efficacy that might arise from strategies that promote mastery over one’s symptoms.

The purpose of the present study was to investigate whether a four-week residential group treatment program for PTSD is associated with improvements in personal wellbeing in addition to symptoms of PTSD. Consistent with findings regarding the similar but distinct concept of quality of life [3], we hypothesized that improvements in personal wellbeing would improve in tandem with improvements in PTSD symptoms. We also wanted to determine to what extent changes in other core overlapping symptom domains (such as anxiety and depression) were associated with overall personal wellbeing. The program includes a 3- and 9-month review of client progress, with the 9-month assessment point being the final scheduled follow-up appointment and the primary endpoint of the present study.

## Method

One hundred and twenty-four participants (Mean age = 45.5 years, *SD* = 10.3; 19.4% female [*n* = 24]) were recruited from an inpatient residential PTSD treatment program between July 2009 and October 2015. All participants had a primary diagnosis of PTSD according to the Clinician Administered PTSD Scale for DSM-IV (CAPS; [10]). Individuals with a current substance use disorder identified either at interview or from the Alcohol Use Identification Test (AUDIT; [11]) are excluded from the treatment program. The study was approved by the St John of God Health Care Human Research Ethics Committee (ref 839).

### Treatment

All participants were attending their first four-week residential group-based treatment program for PTSD. The majority of group participants were funded to attend the program through worker’s compensation claims (57.9% who were mostly former emergency services workers), by the Department of Veteran’s Affairs (27.8%) or the by Australian Defence Force (7.9%), with only a small minority supported through private health insurance (6.3%).

The group program (five days per week for four weeks) included the following components: (i) psychoeducation about PTSD, (ii) arousal reduction strategies, (iii) cognitive restructuring, (iv) exploration of trauma themes such as safety, trust, and power/control consistent with cognitive processing therapy interventions [12] and (v) discharge planning. Concurrent with the group intervention, participants also attended individual therapy sessions twice a week where prolonged imaginal exposure therapy was conducted. The program included a 3- and 9-month review of client progress.

### Measures

The following self-report measures were administered.

The 42-item version of the Depression Anxiety Stress Scales (DASS-42; [13]) was administered. The DASS-42 have been shown to have good internal consistency (Cronbach α’s ranging from 0.89 to 0.96; [14]), strong convergent and discriminant validity [15], as well as favorable test-retest reliability [14].

The Posttraumatic Stress Disorder Checklist for DSM-IV Civilian version (PCL; [16]) was used to assess PTSD symptoms. The PCL is a 17-item measure of current PTSD symptoms. It is strongly correlated with interview-based measures of PTSD, is able to effectively discriminate those with and without a PTSD diagnosis [16] and appears to have sound internal consistency and test-retest reliability [17]. The internal consistency (Cronbach’s α) for the PCL in the present sample was 0.91.

The Personal Wellbeing Index (PWI) is a 7-item scale [6]. The items include standard of living, personal health, life achievement, personal relationships, personal safety, community connectedness and future security. It has an eighth item pertaining to satisfaction with one’s spirituality or religion; however, completion of this item is optional, thus total scores for the present study were derived from summing the first seven items. Each item is rated on a 10-point scale ranging from “No satisfaction at all” (0) to “Completely satisfied” (10), such that higher scores reflect greater levels of personal wellbeing. The scale has sound convergent validity with similar measures of wellbeing [18]. The mean score on the PWI in a large Australian community sample was 75.3 (for the 7-item version; [19]). The internal consistency (Cronbach’s α) for the PWI in the present sample was 0.84.

Other self-report measures are routinely administered as part of an accreditation process for the program. However, these measures were not relevant to the current research question and so are not reported here.

### Data analysis

Descriptive statistics (frequency, means, and standard deviation) were calculated for all key variables using SPSS 24.0. Repeated measures ANOVAs withpost-hoc pairwise t-test comparisons (using a Bonferrroni adjustment) were used to determine which variables changed significantly across time.

Generalized Estimating Equations (GEE) were used to determine which variables predicted PWI total score. The GEE approach allows estimation of regression coefficients that reflect the longitudinal relation between a predictor variable and an outcome variable [20]. In contrast to linear mixed model approaches, the GEE approach makes fewer assumptions about the data. For instance, the GEE approach depends only on correct specification of the mean of the outcome (given the covariates), not necessarily on the joint distribution of both observed data and random effects (as a linear mixed model approach does; [21]). The GEE approach also allows inference at the population rather than individual level. Gender was included as a factor and age, PCL total score, and DASS Depression, Anxiety and Stress subscales included as covariates. Gender was included on the basis that women with PTSD report different trauma histories to those experienced by men and given that PTSD appears to impact the quality of life of women in potentially different ways [22]. Age was included on the basis that some domains of wellbeing, such as perceptions of life achievement, could conceivably be related to age. In accordance with the recommendations of Twisk [20], we ran GEE models both with and without time point in the model, but we discuss findings for the model that includes time given that time point can potentially confound the relation between time-dependent covariates and PWI score.

Goodness of fit tests are not available for GEE analyses; however, comparison of the Quasi likelihood under Independence model Criterion (QIC) values were compared for three correlation structures: unstructured, exchangeable and AR(1), with AR(1) providing the lowest value (29,938.36). Thus, results for the exchangeable correlation structure are presented.

## Results

Data were available for *N* = 124 at the start of treatment; *n* = 115 (91.9%) participants at the end of residential treatment and for *n* = 80 (64.5%) at the 9-month follow-up. Participants who did and did not complete the posttreatment and 9-month follow-up questionnaires did not differ significantly on any demographic variable or on pretreatment PCL, DASS Anxiety, DASS Stress or PWI scores. DASS Depression scores were an exception, with lower pretreatment DASS Depression scores for those with complete pretreatment data at posttreatment (Means = 22.0 [SD = 9.6] vs 30.9 [SD = 7.2); *t* = 2.85, *df* = 122, *p* = 0.005) and 9-month follow-up (Means = 20.9 [SD = 9.47] vs 26.07 [SD = 9.47]; *t* = 2.92, *df* = 122, *p* = 0.004) respectively.

Pretreatment mean PWI total scores (27.85) were well below the mean of the broader Australian community (75.3 for the 7-item version; *t* = 48.3, *df* = 123, *p* < 0.001; [19]) indicating lower overall perceived personal wellbeing.

There was a significant main effect of change in PWI scores across the nine months although the magnitude of improvement was small (*F* (3, 228) = 5.11, *p* = 0.002, η_p_^2^ = 0.06). The only significant pairwise comparison indicated an improvement in PWI scores from pre to posttreatment (from a mean of 27.85 to 32.27; *p* < 0.0001).

Table 1 summarizes the scores on other key questionnaires at pretreatment, posttreatment, 3-month and 9-month follow-up. There were significant main effects for DASS Depression (*F*(3, 219) = 12.45, *p* < 0.0001), DASS Stress (*F*(3, 216) = 11.27, *p* < 0.0001) and PCL (*F*(3, 222) = 9.40, *p* < 0.0001) scores. The significant decrease in PCL scores from pre to posttreatment indicates that the active phase of treatment was beneficial in reducing PTSD symptoms (from a mean of 62.78 to 56.93; *p* < 0.0001). However, across the follow-up period, from posttreatment to 3-month and 9-month follow-ups respectively, PCL scores remained consistent and did not appear to change further from posttreatment levels (all pairwise *p*’s > 0.05). The DASS Depression and Stress subscale scores also improved from pretreatment to posttreatment (from a mean of 20.66 to 16.12, *p* < 0.0001 and 26.71 to 21.26, *p* < 0.0001 for DASS Depression and Stress respectively). However, both DASS Depression and DASS Stress scores *increased* from posttreatment to the 3-month (from a mean of 16.12 to 21.14, *p* < 0.0001 for DASS depression; 21.26 to 25.62, *p* < 0.0001 for DASS Stress) and 9-month follow-ups respectively (from a mean of 16,12 to 21.93, *p* < 0.001 for DASS Depression; 21.26 to 25.96, *p* < 0.0001 for DASS Stress). There were no significant changes in DASS Anxiety scores across the nine months of the study and all pairwise comparisons were not significant.

**Table 1:** Means (SD) for symptom measures and perceived wellbeing at pretreatment, posttreatment, 3-month and 9-month follow-up

	(a) Pretreatment	(b) Posttreatment	(c) 3-month follow-up	(d) 9-month follow-up	Pairwise significant differences^a^
	Mean (*SD*)	Mean (*SD*)	Mean (*SD*)	Mean (*SD*)	
DASS 21 – Depression	20.66 (9.48)	16.12 (9.42)	21.14 (10.24)	21.93 (10.08)	a > b, b < c, b < d
DASS 21 – Anxiety	17.00 (8.42)	15.78 (8.22)	16.75 (8.09)	17.16 (8.65)	ns
DASS 21 – Stress	26.71 (9.38)	21.26 (8.83)	25.62 (8.43)	25.96 (7.98)	a > b, b < c, b < d
PCL	62.78 (11.59)	56.93 (11.66)	56.50 (12.77)	57.31 (11.72)	a > b, a > c, a > d
PWI	27.85 (11.70)	32.27 (11.12)	29.88 (11.92)	30.49 (12.43)	a < b

*DASS 21* Depression and anxiety stress scales – 21 items, *PCL* Posttraumatic Stress Disorder Checklist for DSM-IV Civilian version

^a^Different subscripts correspond to *p* < 0.05 following Bonferroni pairwise corrections for post-hoc comparisons

### Generalised estimating equations (GEE) analyses

The results of the GEE analyses are summarized in Table 2. PCL total scores, DASS Depression, and age were each significant predictors of PWI scores. Decreases in PCL total and DASS depression were significant predictors of increased (improved) PWI scores as well as (older) age. When controlling for other variables, time point was not a significant predictor of PWI scores. We note that the overall pattern of results was the same when timepoint was (Table 2) and was not included in the model (Additional file 1: Table S1) supporting the role of assessed change in other variables as accounting for the observed change in PWI scores.

**Table 2:** Generalized Estimating Equations (GEE) results (*N* = 124)

			95% Wald confidence interval	
	*B*	Std Error	Lower	Upper
Intercept	45.90	3.60	38.85	52.94
PCL total	−0.26	0.05	−0.37	− 0.16
DASS Depression	−0.39	0.08	−0.53	− 0.24
DASS Anxiety	−0.06	0.10	−0.26	0.13
DASS Stress	−0.04	0.08	−0.20	0.12
Age	0.19	0.06	0.07	0.30
Timepoint 4 (9mth follow-up)	1.00	1.06	−1.08	3.09
Timepoint 3 (3mth follow-up)	−0.33	1.03	−2.34	1.70
Timepoint 2 (posttreatment)	−0.27	0.95	−2.13	1.59
Timepoint 1 (Index: pretreatment)	0			
Gender (Male)	−0.81	2.36	−5.42	3.81
Gender (Index: Female)	0			

*DASS* Depression and Anxiety Stress scales, *PCL* Posttraumatic symptom checklist for DSM-IV

A greater PWI score reflects a greater level of personal wellbeing

Given that there is some degree of overlap between two of the items of the PWI (pertaining to perceived safety and future security) and PTSD symptoms, we re-ran the analyses after excluding these items, noting that the internal consistency of the PWI remained high at 0.77, 0.82 & 0.87 at pretreatment, posttreatment and 9-month follow-up, respectively. The overall pattern of results for the GEE analysis was the same in that changes in PCL, DASS-Depression and age each independently predicted changes in PWI scores.

## Discussion

There are three key findings from the present study. First, individuals seeking treatment for PTSD reported low levels of personal wellbeing. This finding is consistent with reports of low levels of life satisfaction among individuals with PTSD [23]. There are also broad parallels with findings from studies of similar constructs in PTSD, such as self-reported health-related quality of life, which have indicated perceived impairments which go beyond psychological symptoms themselves.

Second, there were significant improvements in PWI scores across the 9-months of the study, although these were only small in magnitude and did not remain significant once changes in other variables (such as PTSD symptoms) were accounted for. This relative persistence of low levels of perceived personal wellbeing may be due to the broad range of challenges faced by participants in our sample who were in many cases transitioning from military and emergency services careers to distinctly different roles. These transitions may impact one’s perceptions of standard of living, life achievement and community connectedness [24, 25], none of which were an explicit focus of the treatment program. Other studies that have investigated the benefits of interventions to boost social connectedness and awareness of positive emotions indicate that personal wellbeing may improve when these are explicit aims for treatment [25].

Third, our results also suggest that improved perceived personal wellbeing is associated with older age, and improvements in PTSD and depressive symptoms. This is consistent with findings regarding the similar construct of quality of life [26]. Given that both depression and PTSD are thought to be characterised in part by distorted cognitions about the self, the world and the future [27, 28], it is not surprising that improvement in depression and PTSD symptoms might be associated with changes in perceived personal wellbeing. For instance, depressed individuals have been demonstrated to hold a pervasive and general negative outlook with a tendency to attribute negative events to stable, internal and global factors [29] which could conceivably influence their ratings of personal wellbeing.

The personal wellbeing of individuals seeking treatment for PTSD may possibly be improved in either of two ways. First, our results suggest that improvements in symptoms of depression and PTSD may independently lead to improvements in wellbeing, albeit, to a small extent. This holds particular promise in the case of depression. As noted, in the present study, depression symptoms intensified preceding the 3 month and 9 month follow-ups: If specific interventions could preempt these setbacks then there may also be associated benefits for personal wellbeing. Second, to the extent that the treatment program focused on improvements in PTSD symptoms, there remains the possibility that additional improvements in personal wellbeing may be achieved by including approaches from personal wellbeing therapy [30], or other adjunctive interventions.

The implications of these current findings need to be considered in light of the study’s limitations. Improvement in PTSD symptoms was not a focus of the present study and the overall symptom-focused outcomes of the treatment program have been reported elsewhere [31]. Nonetheless, the relatively small clinical magnitude of improvement in PTSD symptoms is noteworthy and may be reflective of the complexity of clients who attended the program in that most had previously not benefitted from treatment in other services. Alternatively, it may indicate that subset of participants did not receive a full course of exposure-based therapy in their individual sessions. Unfortunately, we lack the data to determine if this was the case. We relied on a single measure of personal wellbeing, albeit one that has been validated in the Australian community and has sound psychometric properties. It may be that measures of Quality of Life across multiple life domains would detect improvements related to more specific areas of daily living. We were also not able to quantify the extent to which the residential treatment program targeted perceptions of personal wellbeing as opposed to PTSD symptoms. The relatively high rate of program participants supported by funding (workers compensation schemes, Department of Veterans’ Affairs and Department of Defence) and the high rate of follow-up attrition (64% of the sample attending the 9-month follow-up) suggests that the findings may not generalize to other studies and settings, although we believe it is reflective of the routine setting in which the study was conducted. Finally, our study did not include a control group or randomized allocation to treatment and control conditions precluding causal inferences about the role of treatment in changes in personal wellbeing.

## Conclusions

Extending the benefits of PTSD treatments beyond a narrow focus on symptomatic improvement is important for helping individuals with PTSD to enhance their lives. The present results highlight how levels of personal wellbeing are typically low in individuals with PTSD and may only improve to a small extent during and following an intensive residential trauma-focused treatment program. Further research should consider the most effective pathways for enhancing personal wellbeing, whether these be by improving symptoms or through approaches that have a separate focus.

## Additional file

Additional file 1: Table 1. Generalized Estimating Equations (GEE) results without time as a predictor (*N* = 124). (DOCX 13 kb)

## Abbreviations

AR: Auto Regressive Order
CBT: Cognitive Behavioural Therapy
DASS-42: Depression Anxiety Stress Scales – 42 item version
GEE: Generalised Estimating Eqs.
PCL: Posttraumatic Stress Disorder Checklist
PTSD: Posttraumatic Stress Disorder
PWI: Personal Wellbeing Index
QIC: Quasi likelihood under Independence model Criterion

## References

[1] Richardson JD, Long ME, Pedlar D, Elhai JD (2008). Posttraumatic stress disorder and health-related quality of life among a sample of treatment- and pension-seeking deployed Canadian forces peacekeeping veterans. Can J Psychiatr.

[2] North CS, Tivis L, McMillen JC, Pfefferbaum B, Cox J, Spitznagel EL, Smith EM (2002). Coping, Functioning, and adjustment of rescue workers after the Oklahoma City bombing. J Trauma Stress.

[3] Schnurr PP, Hayes AF, Lunney CA, McFall M, Uddo M (2006). Longitudinal analysis of the relationship between symptoms and quality of life in veterans treated for posttraumatic stress disorder. J Consult Clin Psychol.

[4] Rapaport MH, Endicott J, Clary CM (2002). Posttraumatic stress disorder and quality of life: results across 64 weeks of sertraline treatment. J Clin Psychiatry.

[5] Ware JE, Sherbourne CD (1992). The MOS 36-item short-form health survey (SF-36): I. Conceptual framework and item selection. Med Care.

[6] International Wellbeing Group (2013). Personal Wellbeing Index.

[7] Keim J, Malesky LA, Strauser DR (2003). Post-traumatic stress disorder (PTSD), life satisfaction and work personality: exploring the relationship with disability. J Appl Rehab Counsel.

[8] Bormann JE, Liu L, Thorp SR (2011). Spritiual wellbeing mediates PTSD change in veterans with military-related PTSD. Int J Behav Med.

[9] Sonne C, Carlsson J, Bech P, Elklit A, Mortensen EL (2011). Treatment of trauma-affected refugees with venlafaxine versus sertraline combined with psychotherapy – a randomsied study. BMC Psychiatry.

[10] Blake DD, Weathers FW, Nagy LM, Kaloupek DG, Gusman FD, Charney DS, Keane TM (1995). The development of a clinician-administered PTSD scale. J Trauma Stress.

[11] Saunders JB, Aasland OG, Babor TF, de la Fuente JR, Grant M (1993). Development of the alcohol use disorders identification test (AUDIT): WHO collaborative project on early detection of persons with harmful alcohol consumption II. Addict.

[12] Resick PA, Monson CM, Chard KM (2017). Cognitive processing therapy for PTSD: a comprehensive manual.

[13] Lovibond SH, Lovibond PF (1995). Manual for the depression anxiety stress scales.

[14] Brown TA, Chorpita BF, Korotitsch W, Barlow DH (1997). Psychometric properties of the depression anxiety stress scales (DASS) in clinical samples. Behav Res Ther.

[15] Antony MM, Bieling PJ, Cox BJ, Enns MW, Swinson RP (1998). Psychometric properties of the 42-item and 21-item versions of the depression anxiety stress scales in clinical groups and a community sample. Psychol Assess.

[16] Blanchard EB, Jones-Alexander J, Buckley TC, Forneris CA (1996). Psychometric properties of the PTSD checklist (PCL). Behav Re Ther.

[17] Wilkins KC, Lang AJ, Norman SB (2011). Synthesis of the psychometric properties of the PTSD checklist (PCL) military, civilian, and specific versions. Depress Anxiety.

[18] Cummins RA, Eckersley R, Pallant J, van Vugt J, Misajon R (2003). Developing a national index of subjective wellbeing:the Australian unity wellbeing index. Soc Indic Res.

[19] Cummins RA, Woerner J, Weinberg M, Collard J, Hartley-Clark L, Horfiniak K (2013). Australian Unity Wellbeing Index: Report 30.0 - The Wellbeing of Australians: Social Media, personal achievement, and work.

[20] Twisk JWR (2003). Applied longitudinal data analysis for epidemiology.

[21] Hubbard AE, Ahern J, Fleischer NL, Van der Laan M, Lippman SA, Jewell N (2010). To GEE or not to GEE: comparing population average and mixed models for estimating the associations between neighborhood risk factors and health. Epidemiology.

[22] Vogt D, Smith BN, Fox AB, Amoroso T, Taverna E, Schnurr PP (2017). Consequences of PTSD for the work and family quality of life of female and male U.S. Afghanistan and Iraq war veterans. Soc Psychiatry Psychiatr Epidemiol.

[23] Karatzias T, Chouliara Z, Power K, Brown K, Begum M, McGoldrick T, MacLean R (2013). Life satisfaction in people with post-traumatic stress disorder. J Ment Health.

[24] Ahern J, Worthen M, Masters J, Lippman SA, Ozer EJ, Moos R (2014). The challenges of Afghanistan and Iraq veterans’ transition from military to civilian life and approaches to reconnection. PLoS One.

[25] Kent M, Davis MC, Stark SL, Stewart LAA (2011). Resilience-orientated treatment for posttraumatic stress disorder: results of a preliminary randomized clinical trial. Journal of traumatic stress. Studies.

[26] Schnurr PP, Lumney CA (2016). Symptom benchmarks of improved quality of life in PTSD. Depres Anxiety.

[27] Beckham EE, Leber WR, Watkins JT, Boyer JT, Cook JB (1986). Development of an instrument to measure Beck's cognitive triad: the cognitive triad inventory. J Consult Clin Psychol.

[28] Foa EB, Ehlers A, Clark DM, Tolin DF, Orsillo SM (1999). The Postraumatic cognitions inventory (PTCI): development and validation. Psychol Assess.

[29] Raps CS, Peterson C, Reinhard KE, Abramson LY, Seligman ME (1982). Attributional style among depressed patients. J Abnorm Psychol.

[30] Fava GA (2016). Well-being therapy: current indications and emerging perspectives. Psychother Psychosom.

[31] Bredhauer K, Anderson R, McGuire A, Warfe P, Waller M, Kanesaraja J. Review of PTSD group treatment programs: phase 2 in-depth quantitative and qualitative analyses. Brisbane: Centre for Military and Veterans’ Health; 2011. Retrieved February 26, 2018 from: https://www.dva.gov.au/consultation-and-grants/research-and-development/health-studies/posttraumatic-stress-disorder.

