# Supplementary material for: Personal wellbeing in posttraumatic stress disorder (PTSD): association with PTSD symptoms during and following treatment
Source: BMC Psychol. 2018 Mar 2;6:7. doi: 10.1186/s40359-018-0219-2 (PMC5833065; doi:10.1186/s40359-018-0219-2)
Supplement: Supplementary file 1 — Table 1. Generalized Estimating Equations (GEE) results without time as a predictor (N = 124). (DOCX 13 kb) [file 40359_2018_219_MOESM1_ESM.docx]

Supplementary Table 1. Generalized Estimating Equations (GEE) results without time as a predictor (*N* = 124).

|  |  |  | 95% Wald confidence interval | |
| --- | --- | --- | --- | --- |
|  | *B* | Std Error | Lower | Upper |
| Intercept | 45.81 | 3.33 | 39.29 | 189.38 |
| PCL total | -0.26 | 0.05 | -0.36 | -0.16 |
| DASS Depression | -0.38 | 0.07 | -0.52 | -0.25 |
| DASS Anxiety | -0.07 | 0.10 | -0.26 | 0.12 |
| DASS Stress | -0.03 | 0.08 | -0.18 | 0.12 |
| Age | 0.19 | 0.06 | 0.07 | 0.31 |
| Gender (Male) | -0.89 | 2.38 | -5.56 | 3.77 |
| Gender (Index: Female) | 0 |  |  |  |

DASS = Depression and Anxiety Stress scales; PCL = Posttraumatic symptom checklist for DSM-IV.

^#^A greater PWI score reflects a greater level of perceived wellbeing.
